# Association of Gut Microbiome and Dipeptidyl Peptidase 4 in Immune-Mediated Inflammatory Bowel Disease: A Rapid Literature Review

**DOI:** 10.3390/ijms252312852

**Published:** 2024-11-29

**Authors:** Sandra F. Gomes, André Valois, Maria Manuela Estevinho, Mafalda Santiago, Fernando Magro

**Affiliations:** 1 Unit of Pharmacology and Therapeutics, Department of Biomedicine, Faculty of Medicine, University of Porto (FMUP), 4200-450 Porto, Portugal; sfferreiragomes@gmail.com (S.F.G.); mmestevinho@gmail.com (M.M.E.); 2 Unit of Medical Education, Department of Public Health and Forensic Sciences and Medical Education, Faculty of Medicine, University of Porto (FMUP), 4200-450 Porto, Portugal; 3 Center for Drug Discovery and Innovative Medicines (MedInUP), University of Porto, 4200-450 Porto, Portugal; 4 RISE-Health, Faculty of Medicine, University of Porto (FMUP), 4200-450 Porto, Portugal; 5 Unit of Clinical Pharmacology, São João University Hospital Center, 4200-319 Porto, Portugal; andreamatovalois@gmail.com; 6 Department of Gastroenterology, Vila Nova de Gaia/Espinho Hospital Center, 4434-502 Vila Nova de Gaia, Portugal; 7 Portuguese Study Group of Inflammatory Bowel Disease (GEDII), 4200-450 Porto, Portugal; mafalda.santiago@gedii.pt; 8 Center for Health Technology and Services Research (CINTESIS), Faculty of Medicine, University of Porto (FMUP), 4200-450 Porto, Portugal; 9 Department of Gastroenterology, São João University Hospital Center, 4200-319 Porto, Portugal

**Keywords:** dipeptidyl peptidase 4, gut microbiome, inflammatory bowel disease, inflammation, immune-mediated inflammatory diseases, immunomodulation, rapid review

## Abstract

Immune-mediated inflammatory diseases (IMIDs) are characterized by dysregulated immune responses and chronic tissue inflammation. In the setting of inflammatory bowel disease (IBD), dipeptidyl peptidase 4 (DPP4) and gut microorganisms have been proved to interplay, potentially influenced by dietary factors. This rapid review aimed to study the DPP4-gut microbiome link in IBD. A search across five databases and two gray literature sources identified seven relevant studies reporting data on DPP4 and gut microbiome in patients with IBD-related IMIDs or in vitro or in vivo models: one cross-sectional, one in vitro, and five in vivo studies. The findings revealed a significant impact of DPP4 and its substrates, i.e., glucagon-like peptide-1/2 (GLP-1/2), on the composition of gut microbiome and on the development of dysbiosis. Increased DPP4 activity is associated with decreased GLP-1/2; increased pathogenic bacterial phyla such as Actinobacteria, Bacteroidetes, Deferribacteres, Firmicutes, Fusobacteriota, Proteobacteria, and Verrucomicrobia; and decreased alpha diversity of beneficial gut microbes, including *Clostridiaceae*, *Lachnospiraceae*, and *Ruminococcaceae* families and short-chain fatty acid-producing bacteria like *Odoribacter* and *Butryvibrio* spp., with exacerbation of intestinal inflammation. This overview revealed that understanding the DPP4-gut microbiome association is critical for the development of DPP4-targeted therapeutic strategies to guarantee gut microbiome balance and modulation of immune response in IBD.

## 1. Introduction

Immune-mediated inflammatory diseases (IMIDs) encompass a spectrum of chronic conditions characterized by aberrant immune responses and tissue inflammation [1]. These conditions pose a significant global health burden, affecting millions of people worldwide with heavy costs for healthcare systems and impact on patient well-being [1,2]. Despite recent medical advances [3,4], the precise mechanisms underlying IMIDs pathogenesis remain elusive, with both innate and adaptive immune responses implicated in disease progression [5,6].

Disruption in the composition and function of gut microbiota (dysbiosis) has been linked to various IMIDs [7], including inflammatory bowel disease (IBD) [8,9,10]. For instance, specific microbial alterations identified in IBD patients include a decrease in bacterial diversity, reduced counts of Firmicutes and Bacteroidetes, and an increase in Proteobacteria [9]. These data result from a broad discussion concerning the importance of gut microbiome in global human health and disease [11,12,13,14], through the production of metabolites and signaling molecules known to interact with the immune system of the host [10,12,13,15,16].

**Figure 1:**
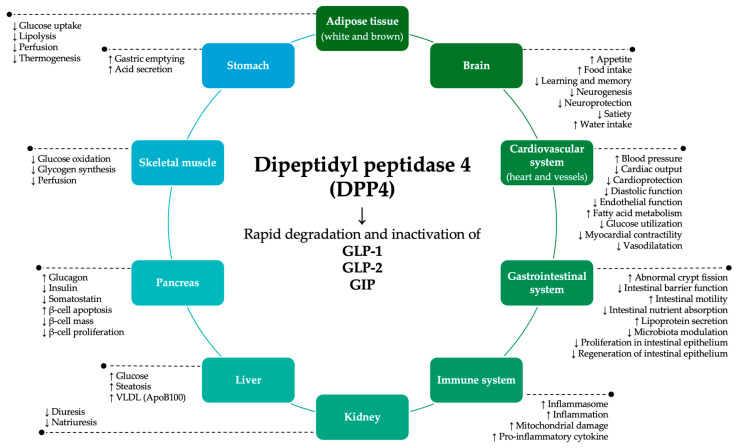
Potential physiopathological functions of DPP4 via degradation and inactivation of GLP-1, GLP-2, and GIP substrates. ApoB100: apolipoprotein B100; DPP4: dipeptidyl peptidase 4; GIP: gastric inhibitory peptide; GLP-1: glucagon-like peptide-1; GLP-2: glucagon-like peptide-2; VLDL: very low-density lipoprotein. Effects of DPP4 activity on physiological functions are represented by arrows: an upward arrow (↑) indicates an increase in the effect, while a downward arrow (↓) indicates a decrease in the effect, based on the reported influence of DPP4 and its substrates.

In this context, dipeptidyl peptidase 4 (DPP4) emerged as a potential link between gut microbiome and IMIDs like IBD [17,18,19,20]. It is a widely expressed membrane-bound glycoprotein available in human serum (sDPP4) and fecal soluble forms (fDPP4) [21]. Fungi and bacteria of the human microbiome (i.e., *Prevotella*, *Lactobacillus*, *Lactococcus*, and *Streptococcus*) also exhibit DPP4-like activity [22,23,24,25,26,27,28,29,30,31,32,33,34]. This serine protease, originally described as cluster of differentiation 26 (CD26), hydrolyzes dipeptides from the N-terminus of polypeptides when a proline or alanine residue is in the penultimate position, thus altering their function [21,35]. DPP4 has an important role in blood sugar regulation by decreasing the bioavailability of glucagon-like peptide-1 (GLP-1), which stimulates insulin secretion and suppresses glucagon release, as well as glucagon-like peptide-2 (GLP-2) and gastric inhibitory peptide (GIP) [36,37]. However, DPP4 acts on a broad range of substrates, from dietary proteins to gut hormones, neuropeptides, and chemokines, with impact on the gut environment, modulating immune responses, among other pathophysiological functions (Figure 1) [17,18,19,20,21,35,36,38,39,40,41].

Despite research advances, the relationship between DPP4 and the gut microbiome in immune-mediated IBD remains unclear and complex. Evidence suggests a bidirectional interaction: changes in the gut microbiome influence DPP4 expression/activity, while DPP4, in turn, modulates the gut microbiome and immune responses [18,19,20,42,43,44]. Further research is warranted to unravel the mechanisms underlying this association and its implications for IBD pathogenesis and potential therapeutic interventions.

This rapid review intended to comprehensively evaluate the current evidence regarding the association of DPP4 and gut microbiome in immune-mediated IBD by exploring the potential influence of DPP4 on gut microbiota composition, development of dysbiosis, and IBD pathogenesis. Understanding these interactions is critical for developing novel therapeutic strategies to restore gut microbiome balance and modulate immune responses in IBD and IMIDs patients.

## 2. Materials and Methods

### 2.1. Protocol, Registration, and Literature Search

This rapid review was conducted in accordance with the Cochrane Collaboration Handbook [45] and followed the Preferred Reporting Items for Systematic Reviews and Meta-Analyses (PRISMA) guidelines [46]. The protocol was developed according to the Preferred Reporting Items for Systematic Review and Meta-Analysis Protocols (PRISMA-P) guidelines [47] and registered in the International Prospective Register of Systematic Reviews (PROSPERO; registration number CRD42023434686).

The literature search was conducted in five databases: CENTRAL (https://www.cochranelibrary.com/central; accessed on 21 July 2023), ClinicalTrials.gov (https://clinicaltrials.gov; accessed on 21 July 2023), PubMed (https://pubmed.ncbi.nlm.nih.gov; accessed on 21 July 2023), SCOPUS (https://scopus.com; accessed on 21 July 2023), and Web of Science (https://webofscience.com; accessed on 21 July 2023), covering all reports published from inception to 21 July 2023. In addition, a gray literature database (Google Scholar; https://scholar.google.com; accessed on 21 July 2023) and reference lists of the included studies and relevant reviews were also approached to ensure the inclusion of all pertinent studies. The search strategy included a combination of controlled vocabulary terms and keywords related to our research question. In brief, queries used for the literature search comprised the intersection of the following main keywords: dipeptidyl peptidase 4, microbiome, and IBD-related immune-mediated inflammatory diseases. The search strategy used in PubMed database is presented in Supplementary Table S1.

### 2.2. Eligibility Criteria and Selection of Studies

Studies were considered eligible for inclusion in this rapid review if they reported data addressing our research question, according to the following PICO elements: (i) population: human subjects with IBD-related IMIDs and in vitro or in vivo models mimicking these diseases; (ii) intervention: not applicable; (iii) comparator: not applicable; (iv) outcomes: DPP4 and gut microbiome data. Due to the limited evidence including our outcomes of interest, no restrictions based on the research subject (i.e., in vitro, in vivo, or human research) were applied to our search strategy.

**Figure 2:**
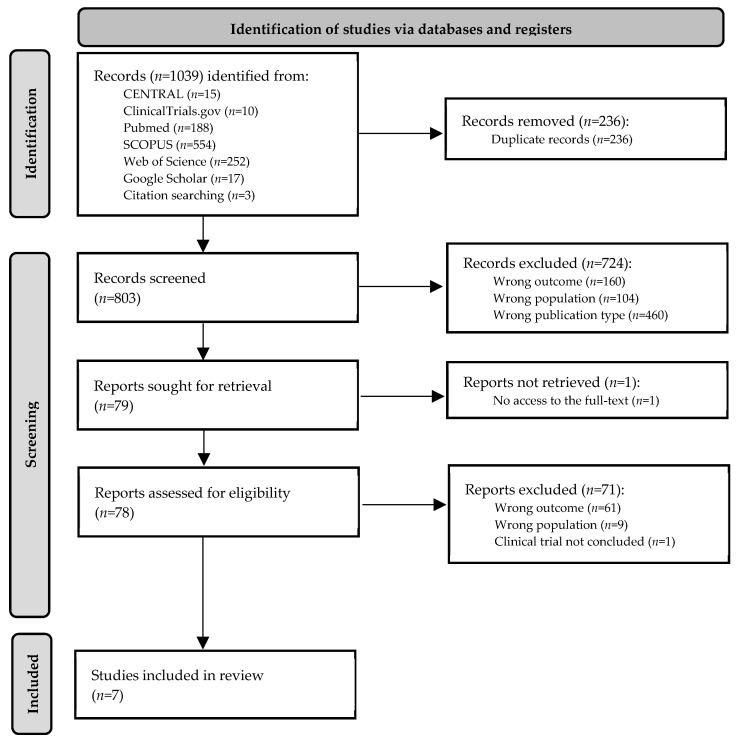
PRISMA 2020 flow diagram.

Studies were excluded if they met any of the following criteria: (i) not relevant to the research question and (ii) the publication type comprises review, systematic review, meta-analysis, book chapter, editorial, letter, or commentary. Article language and geographical localization were not considered as exclusion criteria. The reasons for exclusion of the ineligible studies were recorded to complete the PRISMA 2020 flow diagram (Figure 2).

Rayyan [48] and Review Manager (RevMan, version 5.4.1, 2020) tools were used for the detection of duplications and selection and management of studies. To assess eligibility for inclusion, two independent reviewers screened the titles and abstracts of the retrieved records. Full-text articles of potentially eligible studies were obtained and further assessed for inclusion. Any discrepancies were resolved through discussion and, when necessary, consultation with a third reviewer.

### 2.3. Data Extraction

Study characteristics and outcome data were collected from the included studies: (i) study details (study reference and origin); (ii) study aim; (iii) materials and methods (study design, sample characteristics, sample size (n), DPP4 assessment details, and gut microbiome analysis techniques); (iv) IBD-related IMID; (v) DPP4 outcomes; (vi) gut microbiome outcomes; and (vii) relevant findings. Given the heterogeneity of the methodologies used in the included studies, a meta-analysis approach was not applicable.

### 2.4. Risk of Bias Assessment

A critical appraisal of the included studies was performed to assess the risk of bias using the following tools: “Animal Research: Reporting of In Vivo Experiments” (ARRIVE) [49] guidelines for the in vivo studies; “STrengthening the Reporting of OBservational studies in Epidemiology” (STROBE) [50] guidelines for the cross-sectional study; and a recently developed quality assessment tool for basic science studies [51] for the in vitro study. Different criteria were independently screened by two reviewers. Discrepancies were addressed through discussion with a third reviewer. A qualitative score (i.e., low, unclear, or high risk of bias) was attributed to each criterion, enabling the estimation of the risk of bias in all included studies regardless of the type of research study.

## 3. Results and Discussion

### 3.1. Studies Characteristics

This rapid review comprehensively evaluated the association between DPP4 and gut microbiome in immune-mediated IBD. The literature search identified 1039 studies from seven different sources; 236 records were removed after duplicates detection. Overall, 803 titles and abstracts were screened by two independent researchers based on the inclusion and exclusion criteria; 724 studies met the exclusion criteria for outcome, population, and publication type reasons, and 79 records were identified as potentially relevant to address the research question. From these, 78 full texts were retrieved and screened to identify the seven studies included in this review. The most relevant characteristics of the included studies are summarized in Table 1. The studies, published between 2018 and 2022, were performed in six different countries (Canada, China, France, Germany, Japan, and Korea). Regarding study design, one prospective cross-sectional study was performed in human patients [52], five in vivo experimental studies included mouse models [18,53,54,55,56], and one in vitro experimental study was performed on commensal bacteria from the human intestinal microbiota [57]. Concerning IMIDs, the included studies were mainly related to inflammatory intestinal conditions, such as IBD. However, primary aims and assessment techniques for the outcomes of interest diverged between studies. DPP4-related outcomes were assessed by molecular methods such as enzyme-linked immunosorbent assay (ELISA) [52,55], V-Plex and MESO QuickPlex systems [57], molecular arrays [56], reverse transcription polymerase chain reaction (RT-PCR) [53,54], and classic standard curve-based methods [18]. Regarding microbiome analysis, sequencing targeting the 16S ribosomal RNA gene variable region was the most common methodology [52,53,55,56,57], but two studies approached microbiome outcomes through direct comparison of fecal content in in vivo models, using fecal transplantation strategies [18,54].

### 3.2. Assessment of DPP4-Related Outcomes

DPP4-related outcomes were differentially assessed in the included studies, integrating data from mRNA expression, protein activity, and protein levels of DPP4 or DPP4 substrates, including GLP-1, GLP-2, GIP, and glucagon (Table 2).

GLP-1 gut peptide secretion was evaluated in intestinal neuroendocrine murine secretin tumor cell line 1 (STC-1) cells exposed to 21 commensal bacterial strains from human intestinal microbiota or butyrate (positive control), revealing a significant induced ability to stimulate the production of GLP-1 in *Roseburia intestinalis* AS6, *Blautia obeum* AS32, and *Parabacteroides distasonis* PF-BaE7, and AS93 bacterial strains in comparison with the GLP-1 production induced by butyrate [57]. Conversely, *Doria formicigenerans* AS168 showed a trend towards increased GLP-1 release, but further studies are needed to confirm statistical significance [57].

Hanawa et al. [53] investigated the effect of the artificial sweetener acesulfame potassium (ACK) on the intestinal mucosa and gut microbiota of mice and reported downregulation of GLP-1 and GLP-2 receptors mRNA expression in ACK-treated mice when compared to controls. This led to potentially worse intestinal inflammation with elevated proinflammatory cytokine expression, damage in the small intestine, and increased intestinal permeability [53].

The studies conducted by Lee et al. [54] evaluated the effect of fecal microbiota transplantation (FMT) on DDP4 expression, using fecal material from metformin-treated mice, in high-fat diet (HFD) and regular diet (RD) groups. DPP4 expression was lower in HFD metformin-treated mice compared with those in the HF diet and regular diet groups. In contrast, GLP-1 expression was higher in HFD metformin-treated mice group [54].

The cross-sectional study included in this rapid review developed by Manka et al. [52] revealed that CD patients exhibit lower GLP-1 levels and increased gut motility compared with healthy subjects.

DPP4 activity was studied by Olivares et al. [18] in the cecal content of gnotobiotic mice colonized with human gut microbiota of a healthy subject and in germ-free mice (GFM). The results showed a significantly higher DPP4 activity in colonized mice and no difference in DPP4 activity and expression, suggesting that the DPP4-like activity produced by gut microbiota was the source of DPP4 activity in colonized mice [18].

Peng et al. [55] reported decreased GLP-1 serum levels in dextran sulfate sodium (DSS)-induced colitis mice. In addition, GLP-1 secretion was stimulated by short-chain fatty acids (SCFAs) via GRP43 and GRP41 activation, and mRNA expression of these receptors was decreased in the DSS-induced colitis model [55]. In turn, when exposed to acetic acid, propionic acid, and butyric acid, primary murine colon epithelial cells exhibited significant stimulation of GLP-1 release. However, after stimulation with feces from control mice and DSS-induced colitis mice, the cells exposed to colitis conditions released less GLP-1 [55].

In a spontaneous model of colitis mice using mucin 2 (Muc2) knock-out (KO) mice (Muc2^−/−^ mice), it was demonstrated that circulating GLP-1, glucagon, and GIP remain unchanged compared with healthy controls [56].

Our analysis highlighted diverse mechanisms through which DPP4 modulates gut physiology and immune response in intestinal inflammatory conditions. The expression, activity, and substrate levels of DPP4 were shown to change in response to various stimuli, including artificial sweeteners [53], metformin treatment [54], and colitis induction [55,56]. Overall, the revised evidence suggested a significant role for DPP4 and its substrates, GLP-1 and GLP-2, in regulating the composition of gut microbiota, in IBD-like conditions [52,53,55,56,57]. Decreased levels of GLP-1 and GLP-2, often caused by elevated DPP4 activity, were correlated with an increased abundance of pathogenic bacteria and a decrease in beneficial microbes [57]. This imbalance is assumed to contribute to intestinal inflammation and disease progression in IBD. DPP4 activity was found to influence the abundance and function of gut microbiota, with implications for intestinal inflammation and barrier integrity, and increased DPP4 activity was associated with dysbiosis [18], evidenced by exacerbated intestinal inflammation and disease severity in IBD. This association suggests that targeting DPP-4 activity could be a therapeutic strategy for managing IBD. Furthermore, genetic variations in DPP4 were shown to be involved in IBD pathogenesis, highlighting the complexity of the underlying processes and the importance of personalized approaches in disease management.

### 3.3. Assessment of Gut Microbiome Outcomes

**Figure 3:**
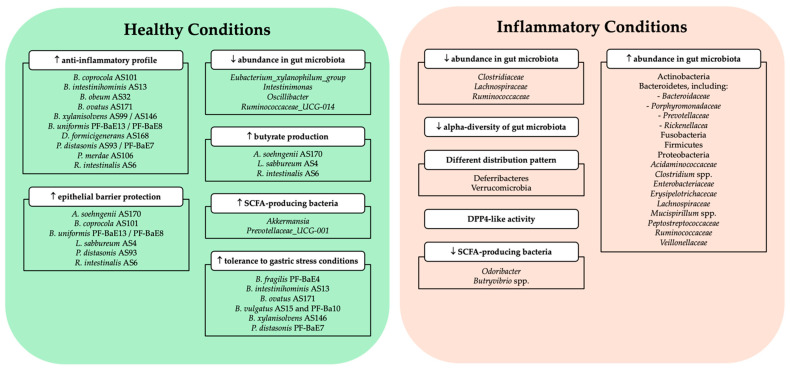
Main gut microbiome outcomes in healthy and inflammatory conditions reported in the included studies [18,52,53,54,55,56,57]. Arrows represent changes in microbiome outcomes: an upward arrow (↑) indicates an increase in the reported outcome, while a downward arrow (↓) indicates a decrease.

The gut microbiome data were analyzed using various methodologies to investigate the association between microbiota composition and both healthy and immune-mediated inflammatory intestinal conditions. Most of the included studies reported data about the diversity and the alterations detected in the composition of the gut microbiota community, ranging from phylum to species level (Figure 3).

Cuffaro et al. [57] revealed that specific bacterial strains exhibit a combination of beneficial functions for a next generation of probiotic candidates: (i) epithelial barrier protection (*A. soehngenii* AS170, *B. coprocola* AS101, *B. uniformis* PF-BaE13/PF-BaE8, *L. sabbureum* AS4, and *P. distasonis* AS93); (ii) anti-inflammatory effect (*B. coprocola* AS101, *B. intestinihominis* AS13, *B. obeum* AS32, *B. ovatus* AS171, *B. xylanisolvens* AS99, *B. xylanisolvens* AS146, *B. uniformis* PF-BaE13/PF-BaE8, *D. formicigenerans* AS168, *P. distasonis* AS93/PF-BaE7, *P. merdae* AS106, and *R. intestinalis* AS6); (iii) butyrate production (*A. soehngenii* AS170, *L. sabbureum* AS4, and *R. intestinalis* AS6); (iv) and tolerance to gastric stress conditions (*B. fragilis* PF-BaE4, *B. intestinihominis* AS13, *B. ovatus* AS171, *B. vulgatus* AS15 and PF-Ba10, *B. xylanisolvens* AS146, and *P. distasonis* PF-BaE7), in addition to the ability to induce GLP-1 production. Among the seven strains that combined two or three probiotic properties, *P. distasonis* AS93 and *R. intestinalis* AS6 exhibited all three functional activities, i.e., barrier protection, anti-inflammatory, and GLP-1 secretion [57]. *B. coprocola* AS101, *B. uniformis* PF-BaE8, and *B. uniformis* PF-BaE13 displayed anti-inflammatory profiles and the capacity to enhance the epithelial barrier [57]. In turn, *B. obeum* AS32 and *P. distasonis* PF-BaE7 exhibited anti-inflammatory properties and the ability to induce GLP-1 production. Overall, only *P. distasonis* PF-BaE7 revealed tolerance to gastric stress conditions [57].

Alpha diversity in gut microbiota was lower in inflammatory intestinal conditions triggered by artificial sweeteners like ACK [53]. In the same study, beta-diversity analysis showed a different distribution pattern in ACK-treated mice at the phylum (differences in the proportion of Actinobacteria, Bacteroidetes, Deferribacteres, Proteobacteria, and Verrucomicrobia), family (increased proportion of *Erysipelotrichacecae* and decreased proportion of *Clostridiaceae*, *Lachnospiraceae*, and *Ruminococcaceae*), and genus levels [53]. The administration of ACK resulted in gut microbiome alterations; however, transferring fecal material from ACK-treated mice to recipient mice did not replicate intestinal damage [53].

In addition, one study showed that the relative abundance of *Akkermansia*, *Bacteroides*, and *Butyricimonas* was not significantly different between HFD and HFD metformin-treated mice [54].

The analysis of the altered gut microbiota composition in CD patients showed that 99% of the total bacterial community belonged to the phyla Bacteroidetes, Actinobacteria, Firmicutes, Proteobacteria, and Fusobacteria [52]. These findings evidenced a lower alpha diversity in gut microbiota of CD patients contrasting with more complex communities in healthy patients [52]. Firmicutes were progressively reduced from control to CD-TNF to CD groups, and Proteobacteria were increased in control and CD-TNF conditions compared with CD group [52]. The most representative families in CD were *Ruminococcaceae*, *Bacteroidaceae*, *Enterobacteriaceae*, *Veillonellaceae*, *Acidaminococcaceae*, *Lachnospiraceae*, *Rickenellacea*, *Prevotellaceae*, and *Porphyromonadaceae* [52]. Also, *Enterobacteriaceae* progressively increased from control to CD-TNF to CD groups, and *Ruminococcaceae* declined when comparing controls with CD-TNF and CD groups [52].

Olivares et al. [18] identified gut microbiota as a source of significant DPP4-like activity in colonized mice, suggesting a functional and structural relationship between DPP4 and DPP4-like activity. DPP4 is a critical enzyme for cleaving peptides with proline or alanine at the penultimate position [21,35]. Interestingly, certain gut bacteria, such as *Prevotella*, exhibit DPP4-like activity (also referred to as PepX activity), which performs similar catalytic functions but originates from microbial sources [18]. These microbial DPP4-like enzymes may complement or modulate host DPP4 activity by influencing the availability of bioactive peptides, potentially impacting protein digestion, gut hormone metabolism, and immune responses [18].

According to the studies performed by Peng et al. [55], the reestablishment of gut microbiota in colitis mice by BLG is associated with an increase in SCFA-producing bacteria (*Akkermansia* and *Prevotellaceae_UCG-001*) and decrease in other bacteria (*Eubacterium_xylanophilum_group*, *Ruminococcaceae_UCG-014*, *Intestinimonas*, and *Oscillibacter*).

The colitis mouse model developed by Ye et al. [56] exhibited a dysbiotic gut microbiome and was characterized by an increased prevalence of several families, including *Prevotellaceae*, *Pophyromonadacea*, *Deferribateraceae*, *Peprostreptococcaceae*, and *Ruminococcaceae*, and genera such as *Clostridium* spp., *Mucispirillum* spp., and *Bilophilia* spp. Furthermore, colitis mice exhibited a reduction in key members responsible for producing SCFAs, including *Odoribacter* and *Butryivibrio* spp. [56].

The assessment of gut microbiome outcomes provided insights into the composition and function of gut microbiota in IBD. Studies revealed dysbiosis characterized by reduced microbial diversity and altered community composition, with distinct shifts in the abundance of key microbial taxa implicated in IBD pathogenesis [52,53,56]. These findings may be explained by the reduction in certain bacterial strains that exhibit probiotic properties, such as epithelial barrier protection, anti-inflammatory effects, and butyrate production, unveiling potential therapeutic approaches for modulating intestinal inflammation and restoring gut homeostasis in IBD [57]. In addition, gut bacteria have been shown to exhibit DPP4-like activity, which may influence the degradation and inactivation of hormones like GLP-1 and GLP-2 in the gut [18]. However, it has not been proven that this bacterial DPP4-activity directly reaches host tissues.

### 3.4. DPP4 and Gut Microbiome: A Dynamic Interplay Shaping Disease Outcomes

The DPP4-gut microbiome relationship represents a critical axis influencing immune homeostasis and inflammatory pathways, particularly in the context of IBD [17,18,19,20]. The modulation of DPP4 directly influences immune pathways by regulating cytokine processing, immune cell activation, and chemokine-mediated migration, thereby shaping inflammatory responses and immune homeostasis [17,18,19,20,21,35,36,38,39,40,41]. Several preclinical and clinical studies show that DPP4 inhibitors (DPP4i) may protect against IBD by modulating T regulatory cells, antigen-presenting cells, cytokines, and GLP-2 [58,59,60,61,62,63,64]. However, clinical findings are inconsistent, with a study indicating increased IBD risk [65] and others finding no association [66,67], suggesting DPP4’s complex and context-dependent role in immune pathways.

The findings from DPP4-related and gut microbiome assessments compiled in this rapid review highlight the bidirectional relationship between DPP4 and gut microbiota in the context of IBD. Higher DPP4 activity has been shown to influence microbial composition by reducing the diversity of beneficial bacteria and increasing pathogenic taxa. Functionally, this microbial imbalance reduces the production of SCFAs, such as butyrate, which are essential for maintaining gut epithelial barrier integrity and suppressing inflammation. Additionally, pathogenic bacteria potentially release proinflammatory molecules (e.g., lipopolysaccharides), which trigger immune responses, promoting cytokine release and exacerbating intestinal inflammation. This microbial dysbiosis reciprocally affects DPP4 expression and activity, creating a feedback loop that perpetuates intestinal inflammation and disease progression in IBD. Furthermore, the review suggests that the development of advanced microbiome-based therapeutics, targeting specific microbial taxa with probiotic properties, constitutes a potential strategy to mitigate intestinal inflammation and restore gut microbiome balance in IBD patients.

### 3.5. Risk of Bias Assessment

The scores (low, high, or unclear risk of bias), attributed for different criteria by each quality tool, are summarized in Supplementary Table S2. Overall, animal studies scored low risk of bias in 56.0% of the applied criteria, but most of the studies were identified with high risk of bias regarding randomization and blinding domains. The cross-sectional study included in this rapid review met 18 out of 22 (81.8%) quality parameters, with a low risk of bias score. Regarding the included basic science study, 64.3% of the applied criteria to assess quality were identified with a low risk of bias score.

### 3.6. Limitations

This rapid review presents limitations that deserve to be discussed. First, the number of included studies is low. In fact, despite the numerous preclinical and clinical studies elucidating DPP4 mechanisms and gut microbiome independently, the published empirical evidence on the association of DPP4 and gut microbiome in the context of IMIDs is scarce. To attenuate this limitation, we adopted an inclusive selection process to collect more comprehensive literature from seven different sources, using an extensive search query, followed by a rigorous screening system based on our research question and inclusion and exclusion criteria. Second, rapid review studies present a risk of bias that cannot be neglected. Our quality assessment attributed “unclear risk of bias” to at least two parameters in each included study and identified the lack of randomization and blinding in animal studies as main methodological limitations. Third, the heterogeneity found in study design, methodologies, and outcome measures hinders broader generalization and comparison of the main findings.

## 4. Conclusions

This rapid review provides a comprehensive overview of the association between DPP4 and the gut microbiome in the context of IBD. The findings suggest that DPP4 plays a pivotal role in modulating immune responses and gut physiology, with its elevated activity linked to dysbiosis, exacerbated inflammation, and disease progression in IBD. Furthermore, the review underscores the bidirectional relationship between DPP4 and the gut microbiota, where DPP4 activity affects microbial composition, and, reciprocally, gut microbiota dysbiosis influences DPP4 expression and function, perpetuating intestinal disease. These insights open new opportunities for therapeutic interventions targeting DPP4, either through pharmacological inhibition or microbiome modulation, to restore gut homeostasis and reduce inflammation in IBD patients. In addition to the potential benefits of using specific probiotics strains to enhance epithelial barrier integrity and modulate immune responses, this review emphasizes the need for developing personalized interventions tailored to patients’ unique DPP4 activity and microbial profile, representing a promising approach for managing IBD. Strategies such as dietary modification and the intentional and conscious use of medicines could complement existing treatments, helping to regulate both microbiome and DPP4 activity to achieve better therapeutic outcomes.

To the best of our knowledge, this is the first attempt to systematically review literature associating DPP4 and the gut microbiome in immune-mediated IBD, offering valuable insights into the bidirectional connection DPP4-gut microbiome, the intestinal disease pathogenesis, and potential therapeutic interventions. However, although some studies demonstrate the potential of DPP4i to reduce intestinal inflammation, its use was also associated with an increased risk of IBD. This conflicting evidence on the effects of DPP4 inhibitors in IBD underscores the need for more targeted investigations to clarify these findings. Further research focused on revealing the cellular dynamics of DPP4 release and shedding, investigating the interplay between microbiota-derived and host DPP4-like activity in translational models, and exploring patient-specific microbiome profiles to inform personalized therapeutic approaches is demanded to elucidate the underlying mechanisms driving DPP4-gut microbiome interactions and validate the therapeutic efficacy of targeting these pathways in the management of IBD and other IMIDs.

## Figures and Tables

**Table 1:** Summary of studies characteristics.

Study Details (Author, Year, and Origin)	Aim	Materials and Methods (Study Design, Sample Characteristics and Size (n), and DPP4-Related Outcomes and Microbiome Analysis Assessment Details)	IMID	DPP4-Related Outcomes	Microbiome-Related Outcomes	Relevant Findings
Cuffaro, 2021 France [57]	Identify and characterize gut bacterial strains with potential health benefits.	In vitro study with the intestinal neuroendocrine murine cell line STC-1 stimulated with human gut commensal strains (n = 21); GLP-1 was quantified using the V-Plex system and MESO QuickPlex SQ 120; Blast comparison of the strain sequence of the V3–V4 variable region of the 16S ribosomal RNA with the NCBI 16S ribosomal RNA sequences database was used to confirm strains.	IBD	- *R. intestinalis* AS6, *B. obeum* AS32, *P. distasonis* PF-BaE7, and *P. distasonis* AS93 induced the production of GLP-1 compared with untreated cells or positive control (butyrate); - *D. formicigenerans* AS168 showed an increase in GLP-1 release compared to unstimulated cells (no statistical significance).	- Seven strains combined two or three probiotic properties: - *P. distasonis* AS93 and *R. intestinalis* AS6 exhibited the ability to strength the epithelial barrier, an anti-inflammatory profile, and the capacity to induce GLP-1; - *B. coprocola* AS101, *B. uniformis* PF-BaE8, and *B. uniformis* PF-BaE13, showed an anti-inflammatory profile and the ability to improve the epithelial barrier; - *B. obeum* AS32 and *P. distasonis* PF-BaE7 presented both anti-inflammatory profile and ability to induce GLP-1; - *R. intestinalis* AS6, *A. soehngenii* AS170, and *L. sabbureum* AS4 produce butyrate and strength the epithelial barrier; - *P. distasonis* PF-BaE7 strain is tolerant to gastric stress conditions.	- Potential health-promoting functions among intestinal commensal strains (7 out of 15 displaying multiple benefits), offering promising candidates for the management of IBD.
Hanawa, 2021 Japan [53]	Investigate the effect of the ACK artificial sweetener on the intestinal mucosa and gut microbiota of healthy mice.	In vivo study with C57BL/6 mice (male, 8 weeks old) treated with ACK; GLP-1 and GLP-2 receptors mRNA expression was assessed by RT-PCR; V4 region of 16S rRNA was analyzed using an Illumina MiSeqIII instrument.	Intestinal inflammatory diseases, e.g., CD	- mRNA expression levels of GLP-1 and GLP-2 receptors were significantly lower in ACK-treated animals in comparison with control animals.	- Alpha diversity of the small-intestinal microbiota in ACK-treated animals was lower than in the control group; - Beta diversity analysis showed a different distribution pattern in ACK-treated mice at the phylum (differences in the proportion of Actinobacteria, Bacteroidetes, Deferribacteres, Proteobacteria, and Verrucomicrobia), family (increased proportion of *Erysipelotrichacecae* and decreased proportion of *Clostridiaceae*, *Lachnospiraceae*, and *Ruminococcaceae*), and genus levels; - ACK treatment brought alterations in the gut microbiome composition, but simply transferring fecal material from ACK-treated mice to recipient mice did not replicate intestinal damage.	- Artificial sweeteners ingestion led to small intestinal damage, increased proinflammatory cytokines, elevated intestinal permeability, and altered gut microbiota composition in mice; - ACK treatment reduced GLP-1 and GLP-2 receptors expression in the intestinal mucosa; - Fecal transplantation from ACK-treated mice did not replicate the small intestinal damage.
Lee, 2019 Korea [54]	Assess how metabolic parameters are influenced by allogeneic FMT.	In vivo study with fecal material collected from metformin-treated C57Bl/6N mice (n = 15, male, 6 weeks old); DPP4 and GLP-1 mRNA levels were quantified by RT-PCR; Microbiome outcomes were a result of the gut microbiome modulation using fecal material from metformin-treated mice.	IBD	- DPP4 mRNA expression was lower in HFD metformin-treated mice group compared with HFD and RD groups; - GLP-1 expression was significantly higher in HFD metformin-treated mice group than in the HFD control group, but not in relation to the RD group.	- Relative abundance of *Akkermansia*, *Bacteroides*, and *Butyricimonas* was not statistically significant between the HFD group and HFD metformin-treated mice.	- FMT led to an increase in the expression of GLP-1; - Reestablishment of gut microbiota and GLP-1 production are potential therapeutic targets in colitis.
Manka, 2021 Germany [52]	Explore the relationship between anti-TNF treatment and hepatic steatosis in CD.	Prospective cross-sectional study with CD patients receiving (n = 18) and not receiving (n = 21) anti-TNF treatment, Infliximab (n = 6) or Adalimumab (n = 12), and healthy controls (n = 10); Serum levels of GLP-1 were quantified by ELISA; Amplicon libraries were analyzed using an Illumina Miseq sequencing platform.	IBD, i.e., CD	- GLP-1 levels are lower in CD than in healthy controls; - Low GLP-1 levels are associated with increased gut motility;	- Lower alpha diversity in gut microbiota of CD patients, compared to a higher community complexity in healthy patients; - In CD, main phyla are Bacteroidetes, Firmicutes, Proteobacteria, Fusobacteria, and Actinobacteria. Firmicutes were progressively reduced from control to CD-TNF to CD, and Proteobacteria were increased in control and CD-TNF compared to CD; - Main families in CD were *Ruminococcaceae*, *Bacteroidaceae*, *Enterobacteriaceae*, *Veillonellaceae*, *Acidaminococcaceae*, *Lachnospiraceae*, *Rickenellacea*, *Prevotellaceae*, and *Porphyromonadaceae*. *Enterobacteriaceae* progressively increased from control to CD-TNF to CD. *Ruminococcaceae* declined when comparing control to CD-TNF and CD.	- GLP-1 is reduced in CD and associated with hepatic steatosis, liver injury, and potentially FXR signaling; - Bowel-movement activity was negatively correlated with GLP-1 levels, suggesting a potential link between gut hormones, bowel activity, and gut microbiota; - Changes in the gut microbiota observed in CD patients.
Olivares, 2018 Germany [18]	Present the characteristics of DPP4-like activity of microbial origin, specify the initial proof of the presence of DPP4-like activity generated by the intestinal microbiota within a living organism, and outline the potential ways in which this microbial DPP4-like activity might theoretically impact the host’s processes of digestion, metabolism, and behavior.	In vivo study with C57Bl6/N GFM (n = 12, male, 4 weeks old) and gnotobiotic mice colonized with the gut microbiota of a healthy subject; DPP4 activity was quantified with a PNA standard curve; Microbiome outcomes were a result of the direct comparison between the cecal content of GFM and gnotobiotic mice colonized with gut microbiota.	Intestinal disorders, e.g., IBD	- DPP4 activity was higher in the cecal content of colonized mice compared to GFM, indicating that the increased activity was due to DPP4-like activity produced by the gut microbiota; - No significant differences in DPP4 activity and expression in the cecal tissue between GFM and colonized mice, suggesting that the microbiota is the source of DPP4 activity.	- Gut microbiota has DPP4-like activity that could potentially impact dietary protein digestion and influence the host’s response to these peptides; - Gut microbiota may modulate host endocrine peptides related to inflammation, metabolism, and behavior.	- Significant DPP4-like activity is present in the gut microbiota, suggesting a novel mechanism through which microbiota may modulate protein digestion, host metabolism, and behavior.
Peng, 2022 China [55]	Study the potential protective effect of the anti-viral traditional Chinese medicine BLG in DSS-induced chronic relapsing colitis C57BL/6 mice.	In vivo study with DSS-induced colitis C57BL/6 mice (male, 6–8 weeks old, n = 3–6); GLP-1 serum levels were quantified by ELISA; V3-V4 region of 16S rRNA genes of distinct regions were analyzed using Illumina Miseq PE300 sequencing platform.	IBD, i.e., UC	- GLP-1 serum levels were decreased in DSS-induced colitis mice; - GLP-1 secretion can be stimulated by SCFAs via GRP43 and GRP41 activation, whose mRNA expression was decreased in DSS-induced colitis; - Exposure to acetic acid, propionic acid, and butyric acid significantly stimulates GLP-1 release from primary murine colon epithelial cells. When exposed to fecal extract from mice with DSS-induced colitis release less GLP-1 compared to control mice.	- Reestablishment of gut microbiota in colitis mice is associated to increasing the abundance of SCFA-producing bacteria (*Akkermansia* and *Prevotellaceae_UCG-001*) and decreasing the abundance of other bacteria (*Eubacterium_xylanophilum_group*, *Ruminococcaceae_UCG-014*, *Intestinimonas*, and *Oscillibacter*).	- Anti-colitis effect of BLG is achieved through the regulation of gut microbiota and reparation of gut SCFA derived-GLP-1 production.
Ye, 2021 Canada [56]	Investigate the impact of intestinal disease on metabolic dysfunction, particularly in the context of IBD, identifying metabolic abnormalities in Muc2^−/−^ mice before the development of severe colitis.	In vivo study with colitis Muc2^−/−^ mice (n = 45); Serum circulating glucagon, GLP-1, and GIP were assessed with a Mouse Metabolic Array; V4-V5 region of 16S ribosomal DNA was analyzed using the Integrated Microbiome Resource.	IBD, i.e., colitis	- Muc2^−/−^ mice exhibited unchanged circulating glucagon, GLP-1, and GIP in comparison with control mice.	- Microbiome of Muc2^−/−^ mice is dysbiotic with shifts in bacterial taxa associated with colitis and enhanced genetic pathways related to lipid metabolism and fatty acid biosynthesis (reduced butyrate levels and increased tendency toward lipid metabolism and lipid biosynthesis pathways); - *Porphyromonadaceae*, *Peptostreptococcaceae*, *Prevotellaceae*, and *Ruminococcaceae*, *Clostridium* spp., *Mucispirillum* spp. are bacterial taxa abundant in human and murine colitis; - Dysbiotic microbiome may be another factor contributing to the metabolic dysfunction comorbid with spontaneous colitis.	- The microbiome in colitis mice displayed dysbiosis. - Glucagon, GLP-1, and GIP seemed unchanged in colitis mice, but metabolic signalling remains associated with dysbiosis.

**Table 2:** Summary of DPP4-related outcomes in the included studies.

DPP4 or DPP4 Substrate	Outcome	Reported Effect *	Reference
DPP4	Protein activity	↑ in the cecal content of mice colonized with human gut microbiota compared with GFM; No significant differences in the cecal tissue of GFM and colonized mice.	[18]
	mRNA expression	↓ in HFD metformin-treated mice compared with HFD and RD control groups.	[54]
GLP-1	Protein levels	↑ in STC-1 cells exposed to *R. intestinalis* AS6, *B. obeum* AS32, and *P. distasonis* PF-BaE7 and AS93 bacterial strains compared with cells stimulated with butyrate or untreated cells;	[57]
		↓ in CD patients compared with healthy controls;	[52]
		↓ in DSS-induced colitis mice in comparison with control mice; ↑ in primary murine colon epithelial cells treated with acetic acid, propionic acid, or butyric acid compared with untreated cells; ↓ in primary murine colon epithelial cells exposed to fecal extract from DSS-induced colitis mice compared to control mice;	[55]
		No significant differences in Muc2^−/−^ mice in comparison with controls.	[56]
	mRNA expression	↑ in metformin-treated mice receiving high-fat diet compared with the high-fat diet group, but not in relation to the regular diet group;	[54]
		↓ in ACK-treated mice in comparison with control animals.	[53]
GLP-2	mRNA expression	↓ in ACK-treated mice in comparison with control animals.	[53]
GIP	Protein levels	No significant differences in Muc2^−/−^ mice in comparison with the control group.	[56]
Glucagon	Protein levels	No significant differences in Muc2^−/−^ mice compared to controls.	[56]

ACK: acesulfame potassium; *B. obeum*: *Blautia obeum*; CD: Crohn’s disease; DPP4: dipeptidyl peptidase 4; DSS: dextran sulfate sodium; GFM: germ-free mice; GIP: gastric inhibitory polypeptide; GLP-1: glucagon-like peptide-1; GLP-2: glucagon-like peptide-2; HFD: high-fat diet; Muc2^−/−^ mice: mucin 2 deficient mice; mRNA: messenger ribonucleic acid; *P. distasonis*: *Parabacteroides distasonis*; RD: regular diet; *R. intestinalis*: *Roseburia intestinalis*; STC-1: secretin tumor cell line 1. * Increased (↑), decreased (↓), or unaltered reported effects are reviewed by DPP4 or DPP4 substrate (GLP-1, GLP-2, GIP, and glucagon) and investigated outcome (mRNA expression, protein activity, and protein levels).

## Data Availability

The authors confirm that the data supporting the findings of this study are available within the article and its Supplementary Materials.

## Supplementary Materials

The following supporting information can be downloaded at https://www.mdpi.com/article/10.3390/ijms252312852/s1.
