# Supplementary material for: Association of Gut Microbiome and Dipeptidyl Peptidase 4 in Immune-Mediated Inflammatory Bowel Disease: A Rapid Literature Review
Source: Int J Mol Sci. 2024 Nov 29;25(23):12852. doi: 10.3390/ijms252312852 (PMC11641704; doi:10.3390/ijms252312852)
Supplement: Supplementary file 1 [file ijms-25-12852-s001.zip › SupplementaryFiles/Supplementary Table S1.pdf]

Supplementary Table S1. Search strategy used for Pubmed database. Query used for the literature search comprised the intersection of dipeptidyl peptidase 4, microbiome, and immune-mediated inflammatory diseases related terms.

**Dipeptidyl peptidase 4**	"dipeptidyl peptidase 4" OR "dipeptidyl peptidase-4" OR "DPP4" OR "DPP 4" OR "DPP-4" OR "dipeptidyl peptidase 4"[MESH] OR "dipeptidyl peptidase IV" OR "DPPIV" OR "DPP IV" OR "DPPR" OR "cluster of differentiation 26" OR "CD26" OR "CD 26" OR "adenosine deaminase complexing protein 2" OR "ADCP2" OR "adenosine deaminase"[MESH] OR "aminopeptidase N" OR "APN" OR "alanyl aminopeptidase" OR "alanine aminopeptidase" OR "AAP" OR "cluster of differentiation 13" OR "CD13" OR "CD 13" OR "glucagon-like peptide 1" OR "glucagon-like peptide 1"[MESH] OR "GLP 1" OR "GLP1" OR "dipeptidyl-peptidase IV inhibitor*" OR "DPP4i" OR "DPP-4i" OR "dipeptidyl-peptidase IV inhibitors"[MESH] OR alogliptin OR "SYR 322" OR "SYR322" OR anagliptin OR bisegliptin OR carmegliptin OR "R1579" OR "RO4876904" OR denagliptin OR "GW 823093" or "GW823093" OR dutogliptin OR "PHX1149" OR evogliptin OR "DA 1229" OR gemigliptin OR "LC15 0444" OR gosogliptin OR "PF 00734200" OR "PF 734200" OR linagliptin OR "BI 1356" OR "BS 1356" OR melogliptin OR "GRC 8200" OR omarigliptin OR "MK 3102" OR sitagliptin OR "MK 0431" OR saxagliptin OR "BMS 477118" OR teneligliptin OR trelagliptin OR "SYR 472" OR vildagliptin OR "LAF 237" OR "LAF237"
**Microbiome**	microbiome OR metabolome OR microbiota OR ecosystem OR flora OR microflora OR dysbiosis OR bacteria OR proteobacteria OR firmicutes OR bacteroidetes OR fungi OR virus
**Immune-mediated inflammatory diseases**	"immune-mediated inflammatory disease*" OR "immune mediated inflammatory disease*" OR "immune mediated inflammatory diseases" OR "IMID*" OR "autoimmune disease*" OR "inflammatory disease*" OR "inflammatory bowel disease*" OR "inflammatory bowel disease"[MESH] OR "Crohn's disease" OR "Crohn disease" [Mesh] OR "ulcerative colitis" OR "ulcerative colitis"[MESH] OR "unspecified colitis" OR "indeterminate colitis" OR "rheumatoid arthritis" OR arthritis OR spondyloarthritis OR "ankylosing spondylitis" OR "connective tissue disorder*" OR vasculitits OR "dermatological disease*" OR "cutaneous inflammatory condition*" OR psoriasis OR "atopic dermatitis" OR "multiple sclerosis" OR "systemic lupus erythematosus" OR sacroiliitis OR "psoriatic arthritis"
