# Supplementary material for: Association of Gut Microbiome and Dipeptidyl Peptidase 4 in Immune-Mediated Inflammatory Bowel Disease: A Rapid Literature Review
Source: Int J Mol Sci. 2024 Nov 29;25(23):12852. doi: 10.3390/ijms252312852 (PMC11641704; doi:10.3390/ijms252312852)
Supplement: Supplementary file 1 [file ijms-25-12852-s001.zip › SupplementaryFiles/Supplementary Table S2.pdf]

**Supplementary Table S2.** Summary of risk of bias of the included studies. Quality assessment of studies was performed with "Animal Research: Reporting of *In Vivo* Experiments" (ARRIVE; Percie du Sert *et al.*, 2020), "STrengthening the Reporting of OBservational studies in Epidemiology" (STROBE: von Elm *et al.*, 2007), and a quality assessment tool for basic science studies (Cosme *et al.*, 2021). Different criteria were scored with low risk of bias ("+", green) if the desirable information was reported in the study, high risk of bias ("-", red) if the criterion was not accomplished through the reported information, and unclear risk of bias ("?", yellow) if the information was not stated or incompletely reported in the study.

**Risk of bias criteria**

*"Animal Research: Reporting of In Vivo Experiments" (ARRIVE) guidelines*

| **Study** | 1 | 2 | 3 | 4 | 5 | 6 | 7 | 8 | 9 | 10 |
|---|---|---|---|---|---|---|---|---|---|---|
| **Hanawa, 2021** | + | ? | ? | - | - | + | ? | + | ? | + |
| **Lee, 2019** | + | ? | ? | - | - | + | + | + | + | + |
| **Olivares, 2018** | + | ? | ? | - | - | + | + | + | + | + |
| **Peng, 2022** | + | ? | ? | - | - | + | + | + | ? | + |
| **Ye, 2021** | + | + | ? | ? | - | + | + | + | + | + |

*"STrengthening the Reporting of OBservational studies in Epidemiology" (STROBE) guidelines*

| **Study** | 1 | 2 | 3 | 4 | 5 | 6 | 7 | 8 | 9 | 10 | 11 | 12 | 13 | 14 | 15 | 16 | 17 | 18 | 19 | 20 | 21 | 22 |
|---|---|---|---|---|---|---|---|---|---|---|---|---|---|---|---|---|---|---|---|---|---|---|
| **Manka, 2021** | + | + | + | + | ? | ? | + | + | + | ? | + | + | + | + | + | + | + | + | + | + | ? | + |

*Quality assessment tool for basic science studies*

| **Study** | 1 | 2 | 3 | 4 | 5 | 6 | 7 | 8 | 9 | 10 | 11 | 12 | 13 | 14 |
|---|---|---|---|---|---|---|---|---|---|---|---|---|---|---|
| **Cuffaro, 2021** | + | + | ? | ? | + | ? | + | ? | + | ? | + | + | + | + |
